# Evaluating the Value of Anti-SARS-CoV-2 Antibody-Based Tests for COVID-19 Diagnosis

**DOI:** 10.3390/jcm11247489

**Published:** 2022-12-17

**Authors:** Xiao-Lu Yu, Jia-Wen Xie, Mao Wang, Mei-Qi Lin, Ya-Wen Zheng, Li-Rong Lin

**Affiliations:** 1 Center of Clinical Laboratory, Zhongshan Hospital of Xiamen University, School of Medicine, Xiamen University, Xiamen 361004, China; 2 Institute of Infectious Disease, School of Medicine, Xiamen University, Xiamen 361004, China; 3 Zhongshan Hospital, Fujian Medical University, Xiamen 361004, China

**Keywords:** COVID-19, SARS-CoV-2, antibody detection, nucleic acid test, diagnosis

## Abstract

Background: The early detection of COVID-19 patients is fundamental for containing the pandemic. A reverse-transcriptase quantitative polymerase chain reaction (RT-PCR), which detects SARS-CoV-2 RNA, is the gold standard diagnostic test, although it can contribute to false-negative results. Consequently, supplementary diagnostic tests are urgently needed. Methods: To assess the value of anti-SARS-CoV-2 antibody-based tests for confirming COVID-19, a retrospective study was conducted on 3120 inbound overseas travelers who underwent a 14-day government quarantine in Xiamen from August 2020 to October 2020. The diagnostic accuracy of the total antibody that detected the anti-SARS-CoV-2 antibody and the RT-PCR that detected SARS-CoV-2 RNA was determined in comparison to the clinical diagnosis. Results: The COVID-19 positive rate was 3.14% (98/3120). The sensitivity and specificity of the RT-PCR test on the first day of quarantine were 14.29% and 100%, respectively, and the sensitivity and specificity of the total antibody were 93.88% and 99.40%, respectively. The kappa value between an RT-PCR on the first day of quarantine and a clinical diagnosis was 0.24 (95% CI, 0.14–0.35), indicating poor consistency. The kappa value between total antibodies and a clinical diagnosis was 0.88 (95% CI, 0.83–0.93), indicating perfect consistency. There were no differences in the positive rates of an RT-PCR in symptomatic COVID-19 (7.41% (2/27)) and asymptomatic COVID-19 (16.90 (12/71) (*p* = 0.338). Similarly, the positive rate of the total antibody tests showed no difference in symptomatic COVID-19 (96.30% (26/27)) and asymptomatic COVID-19 (92.96% (66/71)) (*p* = 0.676). Conclusion: SARS-CoV-2 antibodies are developed by the body in response to an infection or after vaccination; this can easily lead to a missed diagnosis. In the context of low sensitivity for an RT-PCR, SARS-CoV-2 antibody detection is an effective adjunct to RT-PCR detection, which can improve the diagnostic accuracy of COVID-19 and provide an effective complement to the false-negative results of an RT-PCR.

## 1. Introduction

Since its emergence in 2019, severe acute respiratory syndrome coronavirus-2 (SARS-CoV-2) has spread rapidly around the world, leading to the coronavirus disease 2019 (COVID-19) pandemic [1]. The early diagnosis and timely isolation of patients is crucial for epidemic prevention and control. Three kinds of diagnostic tests are pertinent to patient administration and pandemic control [2]: a reverse-transcriptase quantitative polymerase chain reaction (RT-qPCR) test, which examines the presence of viral nucleic acids [3]; a rapid antigen test, which examines viral proteins [4]; and a serology test, which detects host antibodies in response to infection. RT-qPCR and rapid antigen tests are used to diagnose acute infection, while serology tests provide indirect evidence of infection and are used to establish a late or retrospective diagnosis [5]. Although specificity and sensitivity are important characteristics of a test, achieving the right infection diagnosis also relies on the time of sampling relative to the stage of infection [6]. SARS-CoV-2 infections are classified as asymptomatic, pre-symptomatic, or symptomatic, and more than 20% of infection transmission can be credited to a person who is asymptomatic or pre-symptomatic [2]. This characteristic implies that symptom-based testing alone is not satisfactory to control the spread of infection. No test is perfect; how to detect SARS-CoV-2 remains a critical element in the global strategy to control COVID-19.

An RT-PCR, which detects SARS-CoV-2 RNA, is the gold standard diagnostic test recommended by the current guidelines. However, many factors, including inappropriate sample-collection techniques, viral burden, time relative to the stage of infection, and sample source, have been reported to affect the accuracy of RT-PCR assays, which could contribute to false-negative results [7,8]. The sensitivity of an RT-PCR increases from 0% on day 1 to 32% on day 4, with a sensitivity of 80% on day 8 after exposure [9]. Antibody testing can identify if a person has previously been exposed to the virus. Zhao et al. found that combining molecular and antibody testing in the second week after symptom onset can increase the rate of COVID-19 case detection by as much as 40% [10]. Consequently, supplementary diagnostic tests are urgently needed [11]. Serological tests that detect anti- SARS-CoV-2 antibodies are incorporated in national guidelines for testing symptomatic and asymptomatic patients as well as contacts of infectious cases [12]. Here, we conducted a cross-sectional study to explore the value of anti-SARS-CoV-2 antibody-based tests for COVID-19 diagnosis in inbound people from August 2020 to October 2020 in Xiamen port. In addition, we also compared the applicated value of the antibody with the nucleic acid test.

## 2. Materials and Methods

### 2.1. Study Population and Ethics Statement

A cross-sectional study was conducted in Xiamen, China from August 2020 to October 2020. A total of 3120 inbound overseas travelers who underwent a 14-day government quarantine were included in the study. The arrival date was considered as day 1. Each participant was placed in a separate room and tested for SARS-CoV-2 via an RT-qPCR and for total antibodies via a chemiluminescence microparticle immunoassay on days 1, 7, and 14. Participants were diagnosed with COVID-19 based on epidemiologic and clinical evidence of infection according to the diagnosis and treatment protocol for novel coronavirus pneumonia (trial version 7) (released on 3 March 2020) [12]. All participants were discharged on day 14 after excluding COVID-19.

This study was approved by the Ethics Committee of Zhongshan Hospital of Xiamen University and followed the ethical guidelines of the Helsinki Declaration. Due to the retrospective and observational nature of the study, the requirement for individual patient consent was waived.

### 2.2. Nucleic Acid Test

Both nasopharyngeal and oropharyngeal swabs were collected from participants, placed in 2 mL of universal transport media, and tested via an RT-PCR using the Daan 2019-nCoV RT-PCR Kit (Daan gene, Guangzhou, China) for ORF1ab and N genes following the manufacturer’s instructions. The threshold cycle values for both ORF1ab and N genes were ≤40 cycles. Samples positive for both genes were considered positive for SARS-CoV-2 RNA. Samples with either ORF1ab or N gene positivity were reexamined, with repeated positivity for the same gene indicating positivity for SARS-CoV-2 RNA.

### 2.3. Total Antibody Testing

Total antibodies against SARS-CoV-2 in plasma samples were detected using the Innovax-2019-nCoV total antibody kit (Xiamen Innovax Biotech Co., Ltd., Xiamen, China) following the manufacturer’s instructions. The kit was developed based on a double-antigen sandwich chemiluminescence microparticle immunoassay, with the receptor-binding domain of the spike SARS-CoV-2 protein as the immobilized and acridinium-ester-conjugated antigen. The antibody titer was calculated based on the cutoff and was recorded as the cutoff index (COI). A COI of <1.00 and ≥1.00 was considered negative and positive, respectively.

### 2.4. Statistical Analysis

The Mann–Whitney U test was used for continuous variables with a skewed distribution, while a χ^2^ test or Fisher’s exact test was used for categorical variables. The consistency analysis of the two methods was classified as nearly perfect (0.81–1.0), substantial (0.61–0.8), moderate (0.41–0.6), fair (0.21–0.4), or poor (0–0.2), according to the consistency of the kappa value. A receiver operating characteristic (ROC) analysis was performed to determine the performance of total antibodies in the diagnosis of COVID-19. A two-sided *p*-value of <0.05 was considered statistically significant. All statistical analyses were conducted using SPSS statistics version 20 (SPSS Inc., Chicago, IL, USA).

## 3. Results

### 3.1. Characteristics of Participants

Among the 3120 participants, 98 (3.14%) were diagnosed with COVID-19. The 98 individuals with COVID-19 were 81.5% (79/98) male and had a median age of 36.0 years (interquartile range (IQR), 29.0–45.0). The 3022 individuals without COVID-19 were 56.3% (1702/3022) male and had a median age of 45.0 years (IQR, 24.0–64.0). The COVID-19 and non-COVID-19 groups exhibited significant differences in gender (χ^2^ = 22.864, *p* = 0.000) and age (Z = −2.803, *p* = 0.005).

### 3.2. Diagnostic Performance of the RT-PCR Test on Different Quarantines for COVID-19

Of the 98 COVID-19 patients, 14 patients had a positive RT-PCR on day 1 of quarantine and 84 patients had a negative RT-PCR; all 3022 non-COVID-19 patients were negative on the RT-PCR. The RT-PCR demonstrated a sensitivity of 14.29%, a specificity of 100%, a positive predictive value of 100%, and a negative predictive value of 97.30% for a COVID-19 diagnosis by using clinical diagnosis as the gold standard. The kappa value between the RT-PCR and the clinical diagnosis was 0.24 (95% CI, 0.14–0.35), indicating poor consistency. The sensitivity of the RT-PCR increased with the quarantine day—71.43% up to day 7 and 85.71% up to day 14. The kappa value between the RT-PCR up to day 7 and the clinical diagnosis was 0.82, and the RT-PCR up to day 14 and the clinical diagnosis was 0.92 (Table 1).

### 3.3. Diagnostic Performance of the Total Antibody Test for COVID-19

**Figure 1:**
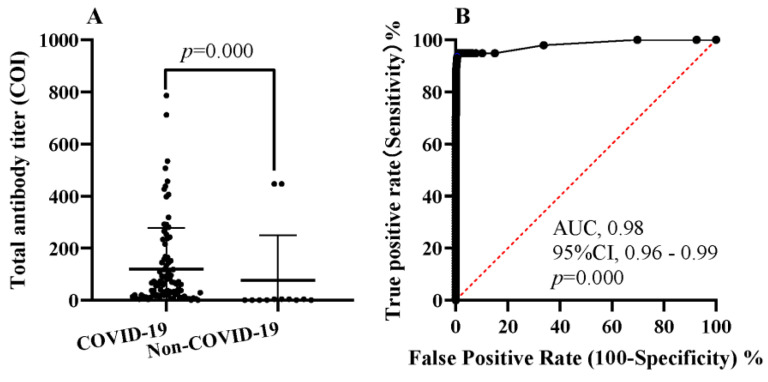
Diagnostic performance of the total antibody test for COVID-19. (**A**) Comparison of the titer of total antibodies in COVID-19 and non-COVID-19 patients; (**B**) ROC showing sensitivity as a function of specificity for the use of total antibodies for COVID-19 diagnosis. COI, the cutoff index.

Of the 98 COVID-19 patients, 92 were positive for total antibodies and the rest were negative; of the 3022 non-COVID-19 patients, 18 were positive for total antibodies and the rest were negative. Excluding those with negative total antibodies (n = 3010), the COI of the total antibodies in the COVID-19 patients was higher than that in the non-COVID-19 patients (62.01 (IQR, 19.88–150.4) vs. 3.09 (IQR, 11.17–4.95), *p* = 0.000) (Figure 1A). Using an ROC curve analysis, the area under the curve (AUC) for total antibodies was 0.98 (95% CI 0.96–0.99) (Figure 1B).The sensitivity, specificity, positive predictive value, and negative predictive value of the total antibodies in the diagnosis of COVID-19 was 93.88% (95% CI, 87.28–97.16%), 99.40% (95% CI, 99.06–99.62%), 83.64% (95% CI, 75.61–89.39%), and 99.80% (95% CI, 99.57–99.91%), respectively. The kappa value between total antibodies and clinical diagnosis was 0.88 (95% CI, 0.83–0.93), indicating perfect consistency (Table 2).

### 3.4. Comparison of the RT-PCR and Total Antibodies for COVID-19 Patients in Different Subgroups

It has been illustrated that the positive rate of the RT-PCR on day 1 had no difference in symptomatic COVID-19 (7.41% (2/27)) and asymptomatic COVID-19 patients (16.90 (12/71) (*p* = 0.338). Similarly, the positive rate of total antibody tests showed no difference in symptomatic COVID-19 (96.30% (26/27)) and asymptomatic COVID-19 patients (92.96% (66/71)), (*p* = 0.676), 96.30% (26/27). The positive rate of total antibodies was higher than that in symptomatic COVID-19 or asymptomatic COVID-19 patients (*p* = 0.000) (Table 3).

## 4. Discussion

COVID-19, caused by SARS-CoV-2, has become a major public problem that threatens human health and safety [13]. Currently, the detection of SARS-CoV-2 RNA using an RT-PCR is the gold standard for diagnosing COVID-19. Different RT-PCR assays have been proposed, though the false-negative rate of RT-PCRs is high due to poor sample collection, inappropriate specimen type, low viral load, variability in viral shedding, and premature testing in the disease process [2]. The results of this study showed that in the first RT-PCR used to diagnose COVID-19 patients, specificity was 100% but sensitivity was 14.29%, and the false-negative rate was as high as 85.71%, similar to previous studies [9,14]. Our study also showed that the early use of nucleic acid testing alone (on the first day of quarantine) may lead to a missed diagnosis. As the number of quarantine days increased, the sensitivity of the RT-PCR was 71.43% up to day 7 and 85.71% up to day 14. The results were similar to Wang’s studies [14], in which they used the same RT-PCR kits and found that the sensitivity of an RT-PCR for a person in quarantine for 7 d, 14 d, and 21 d was 72.25%, 84.59%, and 91.91%, respectively.

In the current international environment, our top priority is rapid screening and diagnosis to find the source of infection, cut off the route of transmission, and reduce the number of infected people. Traditional serological tests can track the virus at a certain stage and have the advantage of high throughput [15], which is good for avoiding the false-negative results that occur with an RT-PCR. Serum antibody detection can be performed based on RT-PCR detection, especially for suspected patients with a long course of the disease and a negative RT-PCR. The simultaneous detection of SARS-CoV-2 antibodies and nucleic acids significantly improves the sensitivity of a COVID-19 diagnosis, even within the first week of onset [10]. Moreover, antibody testing can be an important addition to diagnosing COVID-19 for 10 days or longer following onset. The results of this study showed that the accuracy of the SARS-CoV-2 antibody test in diagnosing COVID-19 was 99.23%, which was higher than that of the first RT-PCR test in diagnosing COVID-19. The sensitivity of total antibody detection was 93.88%, while that of RT-PCR was only 14.29% on the first day of quarantine. Antibody detection made up for the deficiency of SARS-CoV-2 nucleic acid detection.

According to the COVID-19 prevention and control protocol (version 6), asymptomatic infected persons are divided into two categories: those who have no symptoms during the whole process and those who are infected after the incubation period and will have some symptoms in the future. Asymptomatic infected people rarely notice that they are unwell; that is only achieved through screening close contacts, epidemiological investigations, and tracing the source and trajectory, and such covert patients can cause new outbreaks [6]. Due to the repeated epidemic situation in China, Shanghai began to control the river on 27 March 2022, and by 7 April 2022, the number of asymptomatic cases in Shanghai reached 101,116 cases. How to identify COVID-19 is an important research question around the world. In this study, we diagnosed patients with symptomatic and asymptomatic COVID-19 infections based on RT-PCRs and antibodies. Among 71 asymptomatic patients, the positive rate of SARS-CoV-2 antibodies was 92.96% (66/71), while the positive rate of RT-PCR on the first day of quarantine was only 16.90 (12/71). According to a previous study, for asymptomatic patients, the interval from day 1 of a positive RT-PCR test to day 1 of a continuous negative test was 1 to 21 days, with 5 asymptomatic patients consistently testing negative on an RT-PCR 1 day after the date of diagnosis [10]. These results suggest that the proportion of patients who did not show any symptoms during the RT-PCR was probably underestimated, simply because of the longer shedding time of the virus in these patients. Antibody tests hold outstanding promise in identifying patients with a SARS-CoV-2 asymptomatic infection and can provide an effective complement to false-negative nucleic acid test results in COVID-19 patients.

The limitations of this study should be stated. Firstly, before widespread vaccination, an antibody for SARS-CoV-2 could be used as an indicator of infection. However, the human body will also produce corresponding antibodies after vaccination, so knowing how to distinguish a natural infection from a humoral immune response caused by vaccination has become an urgent problem to be solved. Secondly, in the manuscript, we only evaluated total antibodies (mainly IgM/IgG) for the diagnosis of COVID-19, although some research has shown no significant difference between the rapid tests used for the detection of IgM and IgG separately and those used for the detection of combined total antibodies (mainly IgM/IgG). In the future, it is better to study the different performance of IgM and IgG for COVID-19 diagnoses.

## 5. Conclusions

In conclusion, SARS-CoV-2 antibodies are developed by the body in response to an infection or following vaccination, which can easily lead to a missed diagnosis. In the context of low sensitivity for an RT-PCR, SARS-CoV-2 antibody detection is an effective adjunct to RT-PCR detection, which can improve the diagnostic accuracy of COVID-19 and provide an effective complement to the false-negative results of an RT-PCR.

## Figures and Tables

**Table 1:** Diagnostic performance of the first RT-PCR test for COVID-19 based on clinical diagnosis.

RT-PCR		Clinical Diagnosis (Gold Standard)		Sensitivity (%) (95% CI)	Specificity (%) (95% CI)	Positive Predictive Value (%) (95% CI)	Negative Predictive Value (%) (95% CI)	Accuracy (%) (95% CI)	Kappa (95% CI)
		COVID-19	Non-COVID-19						
Day 1	Positive	14	0	14.29	100	100	97.30	97.31	0.24
	Negative	84	3022	(7.36–21.21)	(99.87–100)	(78.5–100)	(96.67–97.81)	(96.74–97.88)	(0.14–0.35)
Up to day 7	Positive	70	0	71.43	100	100	97.58	99.10	0.82
	Negative	28	3022	(61.81–79.43)	(99.87–100)	(94.80–100)	(96.68–98.36)	(98.77–99.43)	(0.76–0.89)
Up to day 14	Positive	84	0	85.71	100	100	99.54	99.55	0.92
	Negative	14	3022	(77.44–91.30)	(99.87–100)	(95.63–100)	(99.23–99.73)	(99.32–99.79)	(0.87–0.96)

**Table 2:** Diagnostic performance of the first total antibody for COVID-19 based on clinical diagnosis.

Total Antibody	Clinical Diagnosis (Gold Standard)		Sensitivity (%) (95% CI)	Specificity (%) (95% CI)	Positive Predictive Value (%) (95% CI)	Negative Predictive Value (%) (95% CI)	Accuracy (%) (95% CI)	Kappa (95% CI)
	COVID-19	Non-COVI D-19						
Positive	92	18	93.88	99.40	83.64	99.80	99.23	0.88
Negative	6	3004	87.28–97.16	99.06–99.62	75.61–89.39	99.57–99.91	98.92–99.54	(0.83 0.93)

IQR: interquartile range.

**Table 3:** The detection of antibodies and RT-PCR tests for COVID-19 patients in different subgroups.

Test	Symptomatic COVID-19 (n = 27)	Asymptomatic COVID-19 (n = 71)	Statistical Analysis *	Statistical Analysis ^#^
	Positive Case n (%)	Positive Case n (%)		
RT-PCR	2 (7.41)	12 (16.90)	χ^2^ = 1.440, *p* = 0.338	χ^2^ = 47.725, *p* = 0.000
Total antibody	26 (96.30)	66 (92.96)	χ^2^ = 0.379, *p* = 0.676	χ^2^ = 82.947, *p* = 0.000

* Comparison of the positive rate of the test in different COVID-19 subgroups. ^#^ Comparison of the positive rate of the different tests for COVID-19 subgroups. RT-PCR, reverse-transcriptase quantitative polymerase chain reaction.

## Data Availability

The datasets generated during the current study are available from the corresponding author on reasonable request.
